# Testicular torsion in an undescended testicle: chasing a diagnosis

**DOI:** 10.1093/jscr/rjac263

**Published:** 2022-06-14

**Authors:** Benedict Reed, Rishi Banerjee, Georgios Tsampoukas, Robert Gray

**Affiliations:** Department of Urology, Stoke Mandeville Hospital, Aylesbury, Buckinghamshire, UK; Manchester University Foundation Trust, Manchester, UK; Department of Urology, Stoke Mandeville Hospital, Aylesbury, Buckinghamshire, UK; U-merge Ltd. (Urology for Emerging Countries), London, UK; Department of Urology, Stoke Mandeville Hospital, Aylesbury, Buckinghamshire, UK

## Abstract

Undescended testicles (UTs) and torsion of the testicle are a rare clinical combination. Symptoms may be misleading and interpreted as signs of other common conditions. Moreover, late identification of an UT may significantly delay the diagnosis and lead to adverse outcomes. Here, we present a case of a 17-year-old boy with cerebral palsy and learning disabilities who presented with painful right-sided inguinal mass. Intraoperatively, he was confirmed to have torsion of an UT and orchidectomy was performed. This article also emphasizes the importance of early diagnosis of testicular ectopia.

## INTRODUCTION

Sudden onset pain and swelling of the scrotum, defined as the acute scrotum, implies an acute pathology of the scrotum and/or its content. Testicular torsion (TT) represents a differential of utmost importance as misdiagnosis results in organ loss, and therefore, scrotal exploration for exclusion is one of the most common procedures carried out on children and young adults [1]. On the other hand, an undescended testicle (UT) is a rare, congenital or acquired condition where one or both testis have incompletely descended from the abdomen through the inguinal canal and are thus absent in the scrotum. Multiple genetic mutations have been found to be associated with maldescent of the testis resulting in ectopia, where the testicle has taken a non-normal path and ended up in unusual, non-scrotal position. A retractile testis, the result of an excessively active cremasteric reflex, resulting in acquired UT has a 32% risk of becoming ascended permanently [2]. The combination of TT and UT is rare—though it is not uncommon for patients presenting with features of torsion to then be diagnosed with an undescended testis. Although causation is not fully clarified, specific genes have also been linked to an increased risk of developing torsion [2]. In cases of undiagnosed UT, implications of the development of torsion are significant as earlier identification could have prevented the need for emergency surgery and possible orchidectomy; risk of future malignancy also needs to be taken into consideration [3].

In this paper, we present a case of a 17-year-old boy with cerebral palsy and learning disabilities who presented with a painful inguinal mass, found to be an UT in torsion. Discussed below is the presentation, diagnostic work-up, intraoperative findings and significance of the case.

## CASE REPORT

**Figure 1:**
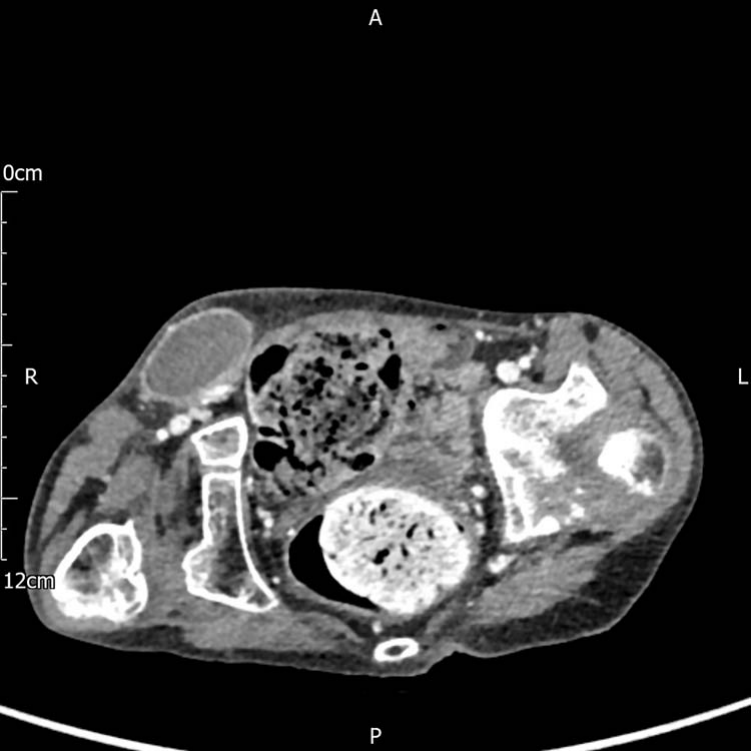
A subcutaneous encapsulated soft tissue mass was described in the right groin raising the suspicion of neoplasia, nodal enlargement or hematoma (axial view).

A 17-year-old boy with a history of cerebral palsy, learning disabilities and asthma who is wheelchair-bound (WHO Performance Status 4) presented with a 24-hour history of general malaise and pain in the right groin. On examination, a swollen and painful, right inguinal mass was detected, and the scrotum was found to be empty and hypoplastic on both sides. The family was reportedly unaware of the possibility of undescended or vanished testicles. The patient was urgently evaluated for a complicated right inguinal hernia and computed tomography (CT) scan of the abdomen (Fig. 1) demonstrated a mass in the right groin that lacked classical radiological features of a hernia.

**Figure 2:**
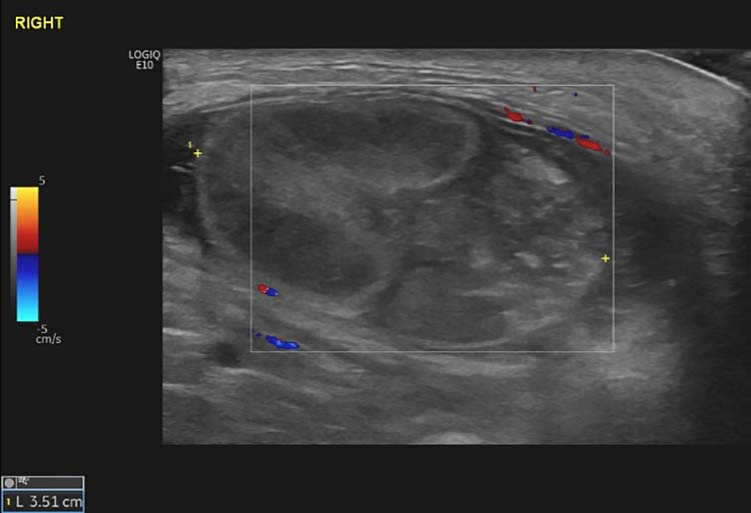
The right testicle was recognized as a 27 × 37 mm heterogeneous mass in the right groin, demonstrating no blood flow. The epididymis was also found bulky. The findings were consistent with the clinical diagnosis of TT in the background of UT.

Subsequent ultrasound of the scrotum (Fig. 2) showed a swollen, heterogeneous and ischemic right testicle that was fixed in the groin suggesting torsion on a background of UT.

The left testicle was not identified. The case was referred to the urology team and urgent exploration was offered considering possible right orchidopexy and/or orchidectomy. The patient underwent surgery later that day. An inguinal incision was made, and the testicle was found to be attached upon the oblique fascia with the gubernaculum attaching superiorly and laterally to the external inguinal ring. The testicle and spermatic cord were dissected from further attachments and the tunica vaginalis was opened. Despite attempts to resuscitate the testicle with warmed gauze and 100% oxygen, its appearance remained severely ischemic and an orchidectomy was performed. The remainder of the procedure was uneventful, and the patient was discharged the next day. The histopathological examination of the specimen revealed hemorrhagic necrosis secondary to infarction—consistent with the clinical history of torsion. The patient was then referred to a specialist for exploratory laparoscopy of the left testicle and to an endocrinologist regarding testosterone replacement therapy.

## DISCUSSION

We consider the above case a key learning point for several reasons. Firstly, the intraoperative findings, the examination of the scrotum and the absence of the left testicle imply that the diagnosis of UT had eluded routine clinical examination for years. If the diagnosis had been achieved earlier, the testicle could have been restored to the scrotal position and this presentation may have been avoided. The above underlines the importance of clinical awareness for the clinicians (e.g. pediatricians, general practitioners) who usually perform routine examinations during early years and of course the education of patients and their guardians [4]. Unfortunately, lack of awareness by healthcare professionals and patients has been noted to be related to delayed attendance and adverse outcomes [5].

Furthermore, the importance of the clinical examination needs to be highlighted. The presence of pain and a mass in the groin region along with an empty scrotum should raise immediate suspicion of a suffering, UT. Proper physical examination contributes significantly to the early diagnosis of intra-abdominal torsion [6]. Despite our case being a complex puzzle due to the vague past medical history, early suspicion could have spared the CT and an ultrasound should have been considered earlier, shortening the time to diagnosis.

The assessment of our patient was made more challenging due to him being non-verbal. The presence of cerebral palsy in combination with learning disabilities has been reported in literature to delay parents seeking help, unfortunately resulting in orchiectomy [7]. In our case, the chief complaint began several hours prior to attendance as malady was not initially clear to his carers. This may have worsened the outcome.

In conclusion, the clinical context of a torsion in the background of UT is associated with adverse outcomes. Salvage rates of the testicle are ~30% as investigation time often exceeds the treatment window for preservation of the organ [8]. The early diagnosis of UT is essential to prevent future complications and when past medical history is unknown, increased clinical suspicion is needed to achieve a prompt diagnosis.
